# Optimal Training Configurations of a CNN-LSTM-Based Tracker for a Fall Frame Detection System

**DOI:** 10.3390/s21196485

**Published:** 2021-09-28

**Authors:** Nur Ayuni Mohamed, Mohd Asyraf Zulkifley, Ahmad Asrul Ibrahim, Mustapha Aouache

**Affiliations:** 1 Department of Electrical, Electronic and Systems Engineering, Faculty of Engineering and Built Environment, Universiti Kebangsaan Malaysia, Selangor 43600, Malaysia; ayuni@siswa.ukm.edu.my (N.A.M.); ahmadasrul@ukm.edu.my (A.A.I.); 2 Centre de Developpement des Technologies Avancees, Division Telecom, Baba-Hassen, Algiers 16303, Algeria; maouache@cdta.dz

**Keywords:** fall frame detection, deep learning, single object tracking, video surveillance

## Abstract

In recent years, there has been an immense amount of research into fall event detection. Generally, a fall event is defined as a situation in which a person unintentionally drops down onto a lower surface. It is crucial to detect the occurrence of fall events as early as possible so that any severe fall consequences can be minimized. Nonetheless, a fall event is a sporadic incidence that occurs seldomly that is falsely detected due to a wide range of fall conditions and situations. Therefore, an automated fall frame detection system, which is referred to as the SmartConvFall is proposed to detect the exact fall frame in a video sequence. It is crucial to know the exact fall frame as it dictates the response time of the system to administer an early treatment to reduce the fall’s negative consequences and related injuries. Henceforth, searching for the optimal training configurations is imperative to ensure the main goal of the SmartConvFall is achieved. The proposed SmartConvFall consists of two parts, which are object tracking and instantaneous fall frame detection modules that rely on deep learning representations. The first stage will track the object of interest using a fully convolutional neural network (CNN) tracker. Various training configurations such as optimizer, learning rate, mini-batch size, number of training samples, and region of interest are individually evaluated to determine the best configuration to produce the best tracker model. Meanwhile, the second module goal is to determine the exact instantaneous fall frame by modeling the continuous object trajectories using the Long Short-Term Memory (LSTM) network. Similarly, the LSTM model will undergo various training configurations that cover different types of features selection and the number of stacked layers. The exact instantaneous fall frame is determined using an assumption that a large movement difference with respect to the ground level along the vertical axis can be observed if a fall incident happened. The proposed SmartConvFall is a novel technique as most of the existing methods still relying on detection rather than the tracking module. The SmartConvFall outperforms the state-of-the-art trackers, namely TCNN and MDNET-N trackers, with the highest expected average overlap, robustness, and reliability metrics of 0.1619, 0.6323, and 0.7958, respectively. The SmartConvFall also managed to produce the lowest number of tracking failures with only 43 occasions. Moreover, a three-stack LSTM delivers the lowest mean error with approximately one second delay time in locating the exact instantaneous fall frame. Therefore, the proposed SmartConvFall has demonstrated its potential and suitability to be implemented for a real-time application that could help to avoid any crucial fall consequences such as death and internal bleeding if the early treatment can be administered.

## 1. Introduction

Activity recognition technology is a crucial component of many higher-level automated systems. This is especially true for systems that require recognition and analysis modules for daily life-monitoring applications. Moreover, intelligent activity recognition systems have been at the forefront of many research topics over the past few years. These systems have been propelled by the advancement of machine learning and parallel computational technologies. Furthermore, the rapid development of micro electro-mechanical systems (MEMS) has led to a renewed interest in sensor technologies for efficient and low-cost embedded sensors. These factors have significantly contributed to the bountiful development of intelligent activity recognition systems particularly in the academic and industrial sectors. Majority of these activity recognition systems were developed to distinguish between typical and atypical human activities [1,2]. Typical human activity is defined as a sequence of typical events within a certain period of time such as walking, standing, or sitting. On the other hand, activities such as falling, slipping, or crouching are usually classified as atypical activities. Activities in the latter group have a similar main trait, and that is the sudden occurrence or drastic change in body movement.

**Figure 1:**
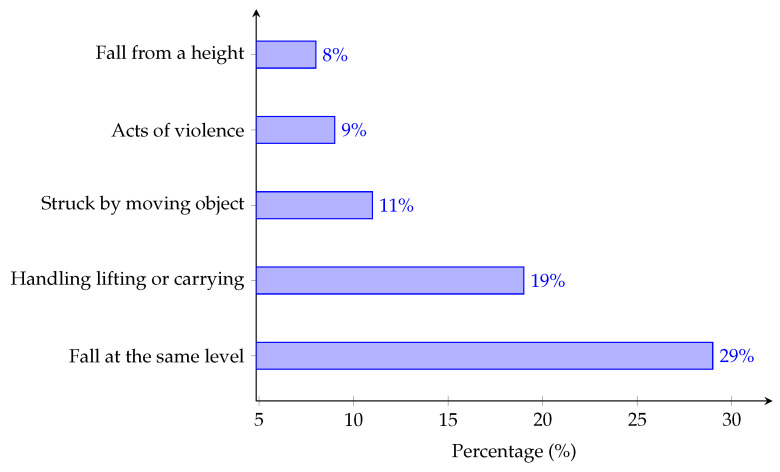
Percentage of non-fatal injuries at the workplace [9,10].

Fall event detection systems are one of the subtopics of activity recognition systems [3,4]. This subtopic has recently gained the attention of many researchers. A systematic review article by the World Health Organization (WHO) [5] in 2014 defined a fall incident as an inadvertent movement that comes to rest on the ground, floor, or other lower level, and does not include an intentional change in position. Likewise, the works in [6,7] have defined a fall incident as a sudden change in body position from either a standing or sitting posture to a lower position as a result of sudden slip or unstable body movements. Moreover, the WHO [8] has also reported that fall incidents are the second cause of death in the unintentional injury category, with an estimation of 646,000 cases each year. Similarly, an article by Reporting of Injuries, Diseases and Dangerous Occurrences Regulations (RIDDOR) in 2019/2020 [9,10] reported that fall incidents constitute the main cause of non-fatal injuries in the workplace, whereby 29% and 8% of fall incidents happened at the same level and from higher places, respectively, as depicted in Figure 1.

In general, a fall incident is recognized as a sporadic event that occurs rarely in comparison to the occurrence of typical human activities. However, unmonitored fall events can pose a serious health risk. These risks may be accompanied by irreversible implications, leading to morbidity and a decrease in the quality of life. Moreover, a fall event can lead to negative consequences for the patient’s financial and psychological states. Ren et al. [11] have further categorized that financial burden can be classified into direct and indirect costs. The direct cost includes the cost of medication, insurance, and rehabilitation. The indirect cost relates to the loss of jobs and/or a decrement in overall working activity. On the other hand, some of the psychological burden examples are traumatic anxiety, fear of falling, and depression, all of which must be taken care of at an early stage. Therefore, an early fall event detection system is crucial to reduce and remove the possibility of negative fall injury-related implications.

Recent research trends have grouped fall event detection techniques based on the type of sensor. The three categories are as follows: wearable devices [12,13,14,15,16], ambient-based devices [17,18,19], and vision-based sensors [20,21,22,23]. Both wearable and ambient-based devices require the sensors to be in close proximity to the targeted users. Commercial wearable devices such as smart watches and smart pendants contain a set of typical sensors of accelerometers, gyroscopes, and barometric pressure. These sensors can be used to detect the fall event. Despite its simplicity, the system requires the users to wear the sensor for a prolonged time. This may cause discomfort to the users, especially to the elderly. Moreover, there are cases in which the elderly may forget to wear these wearable devices that can hinder continuous monitoring [24]. This is a crucial design aspect in elderly monitoring systems as quite a large number of them are suffering from Alzheimer’s disease. Furthermore, one major drawback of this approach is the constant battery supply [25]. Most wearable devices depend solely on the battery supply to operate, which may hamper its operation once the battery is depleted. For example, the Apple Watch SE smartwatch that provides fall detection settings can only run for 18 h after fully charging. Moreover, the adoption of wearable devices is still considerably low for the least-developed countries such as Bangladesh, Cambodia, and Timor-Leste. For instance, Debnath et al. [26] in their article mentioned that the high cost of wearable devices has become the main factor in the low adoption of wearable devices in Bangladesh. Therefore, fall detection through the implementation of wearable devices is hard to be successfully implemented in less-developed countries as many citizens are worried about the maintenance cost. Meanwhile, an ambient-based device uses a set of sensors that include acoustic, infrared, and piezoelectric sensors. However, this approach requires a considerable number of sensors to be installed in order to cover a certain area, resulting in a considerably high initial installation cost. Most of these ambient-based systems can only monitor one person at a time. Moreover, the most noticeable issue in both wearable and ambient-based devices is background noise that profoundly affects the quality of fall detection.

Due to these limitations, many researchers have proposed an intelligent vision-based approach. This method can detect the occurrence of fall events without the use of wearable sensors or sensor meshes. A vision-based system relies on surveillance cameras, such as closed-circuit television (CCTV) or Internet Protocol (IP) cameras, to provide continuous images for activity monitoring. In vision-based systems, artificial intelligence techniques are incorporated through image pre-processing modules to extract the relevant and meaningful features that enable more accurate fall detection. It is worth mentioning that vision-based systems also suffer from background noise. However, the evolution of artificial intelligence technologies has positively influenced these systems, making them more robust to the presence of background noise. Moreover, with the arrival of the 5G era, a faster data transfer rate can be achieved between the cameras and central processors. This improvement in technology leads to better detection accuracy. Henceforth, a vision-based system is more practical in providing a non-intrusive solution with robust detection capability that does not require any wearable devices and complementary ambient sensors [27]. Nowadays, many developing countries have equipped their medical facilities and residential areas with CCTV systems. Therefore, it is a tremendous advantage to utilize these CCTV systems in fall detection applications, mainly to monitor the elderly or people who live alone. Once installed, the vision-based system will become part of their home, and the users do not worry much about the hardware. Furthermore, the camera will monitor the users in a passive mode to detect any fall occurrence. Henceforth, these advantages may complement the drawbacks of wearable devices. Despite its advantages, vision-based systems still suffer from a privacy point of view, which is closely related to the ethical issues that arise from users’ privacy infringement in their living space, such as bedrooms or kitchens [28]. The users need to be fully aware of the situation; as such, the recorded video must not be made available to any third party. The vision-based systems may also trigger a false alarm if the camera is mounted too far from the surveillance area. This situation usually manifests when the system encounters difficulty in detecting the fall event due to weakness in pre-processing modules such as the foreground segmentation process [29].

Nowadays, the deep learning approach has gained a lot of attention in various fields. This is due to the higher level of performance in comparison to standard machine learning techniques [30]. The deep learning method attempts to mimic the human brain mechanism with its multiple layers of artificial intelligence nodes [31]. These nodes subsequently generate automated predictions. The most popular method in the deep learning field is CNN [32]. Due to its capability to extract optimal low-level and high-level features from a set of data [33,34], CNN has been vastly explored in the context of numerous computer vision applications. These applications include object detection, event recognition, semantic segmentation, and human behavioral analysis. However, the usage of CNN for visual object tracking applications has drawn relatively little attention due to the difficulties in fitting the model using limited training data. This is due to the fact that ground-truth information, such as the position and size of the tracked object, can only be extracted from the first frame for a model-free tracker setting. Moreover, a CNN method requires a longer training time, which makes it less practical for online learning-based model updates [35]. These circumstances and challenges have reduced the effectiveness of CNN in object tracking applications. However, the works in [36,37] have designed a compact CNN architecture for their object trackers, namely TCNN and MDNET-N, which train and update the model appearance by using information from the first frame. In contrast to traditional methods [38], where the spatial relationship is the core in generating the object trajectory, the CNN approach emphasizes visual similarity more. These trackers even managed to achieve top performance in the Visual Object Tracking (VOT) Challenge in 2016. The introduction of these compact trackers has become the driving factor for the usage of CNN in fall event detection. On the other hand, LSTM is another deep learning architecture that is used to model the temporal information for the time-series data. LSTM is an improved version of the Recurrent Neural Network (RNN), which is capable of learning the temporal patterns in long-term dependencies. This will alleviate the vanishing gradient problem by incorporating the memory or cell state that is continuously updated using non-linear gating functions. The forget gate deletes the irrelevant information from the previous time step, while the input gate allows only the significant information to be used for the update of the memory cell. Lastly, the output gate will decide which of the information is relevant enough to be a part of the output. The superior capabilities of LSTM have been recognized and implemented in several applications, such as machine translation [39,40,41], natural language processing [42,43,44], and time series analysis [45,46,47,48]. In this manuscript, the capabilities of both CNN and LSTM are motivating factors in our choice to employ both architectures for fall detection.

In addition, most of the vision-based fall detection systems [49,50,51,52,53] that adopt CNN are mainly developed to classify a fall event from typical human activities without identifying the exact instantaneous fall frame. The exact instantaneous fall frame is regarded as the frame at which the fall event starts to happen. Most recent systems only detect a fall event after the patient has fallen and remained on the floor for a considerable time, which is around 10 s for most of the systems. It is crucial to know the exact instantaneous fall frame as it dictates the system’s response time. For example, if a patient has fallen in the restroom, the possibility of a drowning case is high, where a faster lead time of 10 s might be able to save their life. The technical note by Kakiuchi et al. [54] mentioned that bath-related drowning is the top death contributor in Yokohama city for the elderly aged 65 and above. In addition, McCall and Sternard [55] stated that usually, it takes around less than 2 min for an adult to lose consciousness in drowning cases. Therefore, a faster response time might save the patient’s life once the caretaker has been notified to confirm the occurrence of a fall event before requesting medical assistance. This is in line with another noticeable drawback of the current vision-based systems. The detection rate is slow, as most patients remain lying down in the lower position for a particular time before they are noticed. Coherently, any crucial fall consequences such as death and internal bleeding can be better mitigated if the fall occurrence can be discovered earlier. Therefore, this paper covers two important goals: (1) to utilize the CNN as a regressor (tracker) instead of classifier in fall event detection, and (2) to detect the presence of fall events based on the exact instantaneous fall frame. Hence, this paper intends to investigate the optimal training configurations of our tracker, namely SmartConvFall, for instantaneous fall frame detection. The proposed tracker’s architecture involves two-stage modules: object tracking and instantaneous fall frame detection. The first module utilizes a fully CNN-based tracker as the backbone to obtain the objects’ trajectories for all of the video sequences. Next, the second module determines the exact instantaneous fall frame by modeling the contiguous objects’ trajectories through the LSTM architecture. Therefore, the key contributions of this paper can be summarized as below:

1. Modification of state-of-the-art CNN architecture, namely VGG-M architecture, to produce a more compact architecture that is suitable for single object tracking applications;
2. The strategy in maintaining the multiple-model object appearance of SmartConvFall that is used to handle model drift and noisy updates;
3. Extensive comparison of training configurations that cover the selection of the optimizer, learning rate, mini-batch size, number of training samples, and region of interest schemes to obtain the best object appearance model for SmartConvFall;
4. Designing a fall detection based on the exact instantaneous fall frame by integration of stacked layers of LSTM architecture to model the objects’ trajectories.

The latter part of this paper is organized as follows: Section 2 discusses the recent works on CNN-based and LSTM-based fall events detection, Section 3 explains in depth the workflow of the proposed SmartConvFall architecture, Section 4 presents the detailed simulation results and discussion on the SmartConvFall performance, and Section 5 summarizes the research contributions and research directions on the future work.

## 2. Related Works

Generally, a vision-based fall detection system can be broadly categorized into either a standard machine learning approach or a deep learning approach. A basic processing pipeline for most of the vision-based systems is comprised of background subtraction, feature extraction, and fall detection. Each step in the pipeline requires hand-crafted features that were designed by researchers as the input to the machine learning models. On the other hand, a deep learning approach allows the network to be trained in an end-to-end framework with minimal pre-processing steps to obtain the optimal features [56]. In recent years, there has been an increasing amount of published research on the development of deep learning models for fall event classification.

One of the earliest fall event classification systems that adopted the CNN methodology was introduced by Yu et al. [57], in which a CNN classifier was used to classify four different classes of human poses (i.e., bending, standing, lying, and sitting). A fall event is then flagged if the proposed CNN model managed to detect a lying posture event in the ground region. Another work by Adhikari et al. [58] has designed a similar approach but with the addition of crawling poses. Moreover, they analyzed different sets of image combinations that include RGB, Depth, RGB-D, and subtracted background of RGB-D for input to a CNN classifier. The best results were obtained using the subtracted background of RGB-D for fall event classification. However, neither of the previous methods were designed to be end-to-end frameworks, in which manually optimized pre-processing modules were employed to extract human silhouette information before they were fed as input to the CNN models. Meanwhile, a great deal of attention was placed on Li et al.’s [59] work, which proposed a fully CNN-based fall classification system. This system used AlexNet configurations with a compact five convolutional layers network. A compact network allows the models to be updated using much less training data [60]. The researchers made some modifications to the original architecture, particularly with the number of hidden nodes used for the last two fully connected layers. They also removed all response normalization layers to ensure the fall events could be classified from the ADL activities. Recently, a conceptual approach by [51] suggested a combined approach of head segmentation and CNN classifier to detect the presence of fall events. The main assumption was that a large sudden change in the head’s amplitude can be observed during a fall event. Additionally, a shallow CNN architecture was proposed with only one convolutional layer to ensure fast training convergence for a real-time application.

Several studies have been conducted using motion information as opposed to color information as the input data to CNN classifiers to detect the presence of fall events. Min et al. [61] devised a motion-based method to detect a fall event that happened on furniture, rather than on typical general surfaces. They employed Faster R-CNN to extract the information on humans and furniture bounding boxes. A fall event is detected by measuring the changes in motion activity characteristics, such as human shape aspect ratio, centroid, and velocity of the subject. Similarly, a systematic approach by Feng et al. [62] has also incorporated Faster R-CNN to extract human bounding boxes in a video sequence. Motion history image (MHI) and histogram of oriented gradient (HOG) were also extracted. These were subsequently fed to the support vector machine (SVM) to detect the occurrence of fall events. Additionally, Marcos et al. [63] incorporated a stack of optical flow images as input to the CNN network for fall event detection. The work in [64] introduced a two-stream fall classification network using VGG-16 architecture as the backbone model. The first stream was dedicated by motion characteristics used to determine the falling behavior, while the second stream finalized the occurrence of fall events using the VGG-16 model. Interestingly, they replaced the traditional convolutional layers of VGG-16 architecture with a depthwise separable convolution operation to produce a lightweight version aimed for mobile applications.

Previous studies have revealed that an RNN has been implemented in the development of fall classification systems. The work by Lin et al. [21] employed the RNN architecture using LSTM as the underlying model to capture temporal relationships of the body joints, which were pre-extracted using OpenPose. LSTM classifies the fall features from the non-fall features based on the change trajectory of the extracted body joint points. Aside from that, the researchers in [22] modeled the temporal information of the successive frames using LSTM to provide a time-based fall classification. The proposed architecture was comprised of a two-stage module. The goal of the first stage was to produce foreground masks using Mask R-CNN. The segmented masks were then fed into a CNN model to extract meaningful features before a fall event was classified. The classification categories include four human poses: forward fall, lateral fall, jumping, and picking up an object. Likewise, a conceptual fall classification through LSTM architecture was also suggested by [23]. However, their fall system was developed using multiple-camera input for multiple-person surveillance applications. Moreover, they introduced a combined network of temporal and spatial information as the input data for their LSTM model. The temporal information includes the angle between the axis of the upper body and the vertical axis, while the spatial information includes the aspect ratio of the bounding box that encapsulated the person of interest. Additionally, a study in [65] employed the transfer learning approach in training their LSTM architecture to compensate for the lack of a large dataset. The training process began by training the multi-class LSTM model using numerous samples of atypical human activities. Then, the pre-trained parameters were frozen and transferred for a two-class LSTM classifier to detect a fall event occurrence.

## 3. Methods

**Figure 2:**
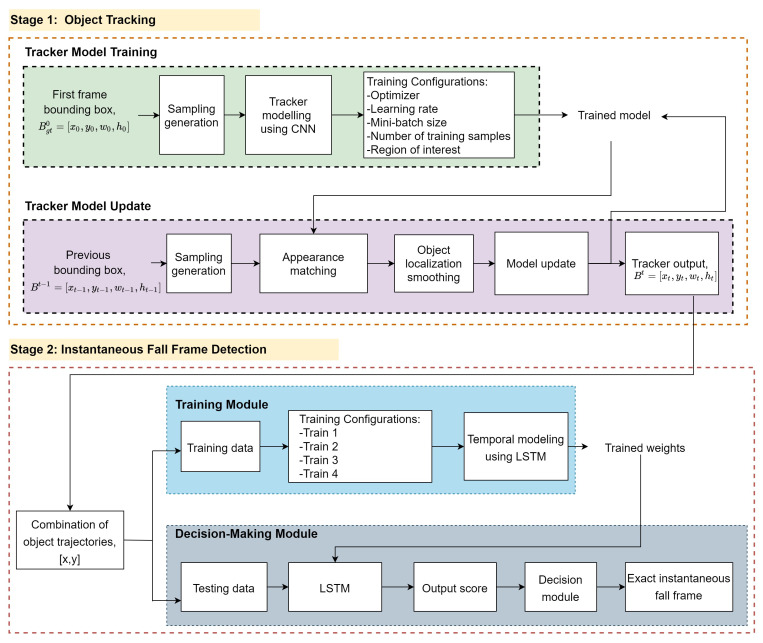
The workflow of the proposed fall frame detection methodology.

The proposed fall frame detection methodology consists of two stage modules, which are object tracking and instantaneous fall frame detection. Figure 2 depicts the full workflow of the proposed SmartConvFall architecture, starting with the training setup of the tracker model until the identification method of the exact instantaneous fall frame. The first stage’s goal is to track the object of interest throughout the whole video sequences using a fully CNN-based tracker. Extensive comparisons of the training configurations covering the selection of optimizer, learning rate, mini-batch size, number of training samples, and region of interests are evaluated to obtain the best-trained model. Meanwhile, the second stage goal is to determine the exact instantaneous fall frame by modeling the temporal information between the contiguous spatial object trajectories using the LSTM architecture.

### 3.1. Dataset

**Figure 3:**
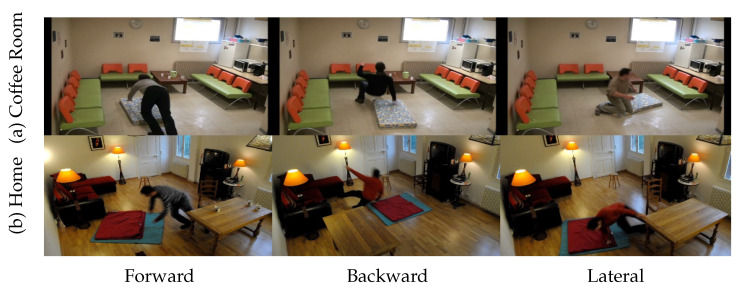
Some image samples for various fall angles in FDD dataset.

In this paper, the Fall Detection Dataset (FDD) [66] that was developed by the Laboratory of Electronics, Information, and Image (Le2i) is used to conduct the experiments. The FDD contains 129 annotated video simulations with 43 videos of Activities of Daily Living (ADL) and 86 videos of fall simulations captured in realistic video surveillance settings. All simulations were recorded using a single camera of frontal view with a fixed frame size of 320 × 240 pixels and a frame rate of 30 frames/s (fps). Moreover, the fall and ADL simulations were simulated by eight different actors in two separate situations, denoted as “Coffee Room” and “Home”. However, this experiment does not utilize the ADL video simulations since our main objective is to determine the fall events only. For fall simulations, each actor is required to perform the ADL simulations, such as standing, sitting, or walking, before performing falling simulations, with an average of 14 fall frames. In addition, the FDD dataset contains various fall angles comprised of forward, backward, and lateral falls due to loss of balance and inappropriate sitting-down situations. All simulated activities are also captured in various directions without considering the camera’s point of view. Moreover, the simulated video also demonstrates the main difficulties of realistic video sequences found in the elderly home environment such as illumination changes, shadows, reflections, occlusions, and cluttered environments. Figure 3 displays some samples for various fall angles captured by different simulation situations.

### 3.2. Stage 1: Object Tracking

**Figure 4:**
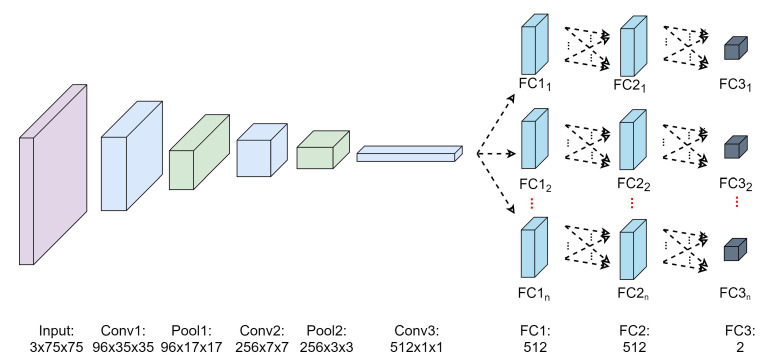
The proposed tracker architecture.

In this module, a fully CNN-based tracker is implemented to track the objects’ trajectories for each video sequence using a network with three convolutional layers and three fully connected (FC) layers as demonstrated in Figure 4. The CNN networks utilize a similar basic configuration of the first three CNN layers from the VGG-M architecture [67]. However, the configurations of the FC layers are modified to produce a compact tracker model suitable for object tracking applications as devised in [68]. Table 1 displays the full configurations of SmartConvFall tracker architecture. The images from the FDD dataset will be down-sampled to 75×75 pixels by linear interpolation before they can be fed to the first CNN layer due to the image setting requirement of the modified VGG-M architecture. The number of convolution filters for the first, second, and third layers are fixed to 96, 256, and 512, respectively. In the meantime, the kernel size of the filters for each CNN layer is set to 7×7, 5×5, and 3×3 pixels, respectively. The number of hidden nodes for both first and second FC layers is set to 512. The last layer consists of a single node to represent two output classes that aim to distinguish the tracked objects and their background. Moreover, all convolutional and FC layers are trained coupled with Rectified Linear Unit (ReLU) activation function; the exception to this lies in the last FC layer, which is coupled with a softmax activation function. Aside from that, the SmartConvFall tracker maintains a set of object appearance models that is useful in resolving the occlusion noise and facilitates re-detection of the tracked object. The illustration of multiple-model object appearance is portrayed as in Figure 4, with *n* referring to the total number of object appearance models.

#### 3.2.1. Sampling Generation

Specifically, the SmartConvFall is designed to be a model-free tracker, in which the object appearance model only received the ground truth annotations during the first frame. Hence, a model-free tracker requires careful model update procedures using a weak supervision approach to distinguish the tracked objects from the background throughout the contiguous video frames with limited prior knowledge. Moreover, the proposed tracker is constructed as a binary classifier, with the tracked object being represented by the positive class, while the background class is set to be the negative class. Therefore, a set of sampling data, $B_{train}$, is extracted to represent both the foreground, $B_{train,+ve}$, and background regions, $B_{train,-ve}$, as follows:

(1) $$B_{train}=B_{train,+ve}⋃B_{train,-ve}$$

The previous bounding box $B^{t-1}=\left[x_{t-1},y_{t-1},w_{t-1},h_{t-1}\right]$ will be used to generate a set of image patches for $B_{train,+ve}^{t}$ and $B_{train,-ve}^{t}$ as laid out in Equations (2) and (3), with *t* representing the current frame and $s_{+ve}$ and $s_{-ve}$ denoting the total number of positive and negative patches, respectively.

(2) $$B_{train,+ve}^{t}=\left\{b_{+ve}^{0},\ldots ,b_{+ve}^{s_{+ve}}\right\}$$

(3) $$B_{train,-ve}^{t}=\left\{b_{-ve}^{0},\ldots ,b_{-ve}^{s_{-ve}}\right\}$$

**Figure 5:**
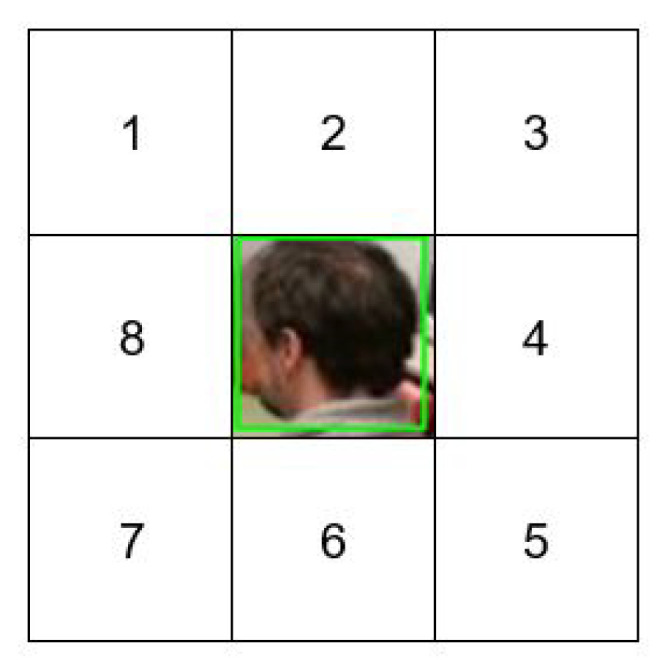
The eight-neighbourhood regions for $B_{train,-ve}^{t}$.

**Figure 6:**
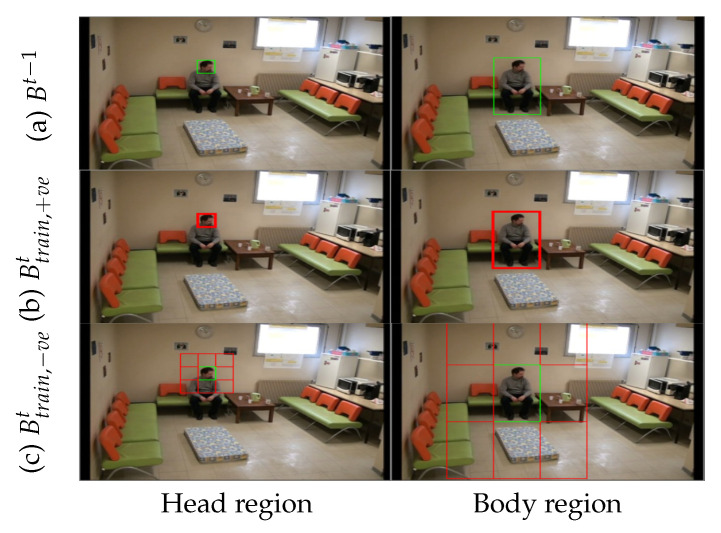
The sampling candidates of $B_{train,+ve}^{t}$ and $B_{train,-ve}^{t}$ for two different region of interest.

Primarily, the image patches for $B_{train,+ve}^{t}$ are generated by sampling the foreground regions that are similar to $B^{t}$ by shifting them with a translation stride, a=10 pixels. The value of *a* is set to a maximum of 10 pixels to ensure each $b_{+ve}$ still contains about 40% of the $B^{t}$ information, which is important in handling the small-scale region of interest. This is due to the smallest width of the bounding box being 18 pixels; hence, if the translation stride is larger than 10 pixels, the $b_{+ve}$ will lose the crucial information of the $B^{t}$. Contrarily, the image patches for $B_{train,-ve}^{t}$ are acquired by sampling background regions that are close to $B^{t}$ by restricting them to eight neighborhood regions as illustrated in Figure 5. Firstly, each of the eight neighborhood regions is discarded if the regions are outside the frame size. Then, all successful regions will be shifted according to the translation strategy similar to $B_{train,+ve}^{t}$. Figure 6 displays some sampling candidates for both $B_{train,+ve}^{t}$ and $B_{train,-ve}^{t}$. The number of sampling candidates for $B_{train}$ will be tested for different values that are set to 400, 600, and 800. This is enforced to ensure that the SmartConvFall is trained using a sufficient number of sampling candidates since the SmartConvFall is a model-free tracker that is highly dependent on the training configurations of the first frame.

#### 3.2.2. Tracker Model Training

The training process of the first frame is indispensable for a model-free tracker, as it can influence the tracker performance in the subsequent frames. Moreover, the CNN networks of the SmartConvFall implement pre-trained VGG-M weights and biases to overcome the issue of insufficient training data. Searching for the best training configurations is a crucial step to obtain the best-trained object appearance model to be used in the entire tracking process. Hence, several hyperparameter settings are optimized, including the optimizer, learning rate, size of mini-batch, and the number of training samples $B_{train}$. These hyperparameters are regarded as the key parameters that need to be emphasized during the training process of the CNN networks. Table 2 summarizes the details of each of the hyperparameters with their respective functions. The first experiment tests five types of optimizers: Adam [69], AdaGrad [70], Stochastic Gradient Descent (SGD) [71], AdaDelta [72], and RMSProp [73]. These optimizers play an important role in updating the model parameters in order to reduce the losses during the training process. As such the training process of the CNN model is based on the backpropagation update. This update aims to minimize the loss function to zero in each iteration. Next, the second experiment investigates the optimal learning rate value used to train the CNN model in the first frame. This is achieved by controlling the step size to update model weights with respect to the loss gradient. Choosing an appropriate learning rate can be difficult, and this is a grave matter of concern. A small learning rate may lead to slow convergence in reducing the loss function, while a large learning rate may hinder convergence and cause the loss function to fluctuate. Therefore, the learning rate values to be tested in this experiment are fixed to 0.0001, 0.001, 0.01, and 0.1. The third experiment inspects the optimal mini-batch size that can be used to train the CNN model. The mini-batch will control the size of training samples for each training iteration. This hyperparameter setting is crucial due to the nature of the CNN model that is based on the backpropagation algorithm. This algorithm estimates the loss function error based on the mini-batch scheme at one time, not the entire training sample. Moreover, the selection of mini-batch size is also constrained by the available computer and graphics processing unit memories. Thus, the size of the mini-batch is only set to 64, 128, and 256. The next experiment evaluates the number of training samples that will be fed into the CNN networks. This experiment is conducted according to the transfer learning approach employed in the SmartConvFall architecture. Finding the optimal number of training samples is indispensable to ensure that the SmartConvFall is able to learn better objectness information of the tracked objects. A number of training samples of 400, 600, and 800 sampling candidates are tested in this experiment. Additionally, the selections of the region of interest between the head and body regions are also carried out to determine the best features to use in the entire tracking process. The comparison between both regions is depicted as in Figure 6. Moreover, each training cycle is set to a maximum of 50 epochs with an equal number of $B_{train,+ve}$ and $B_{train,-ve}$. Additionally, both CNN and FC layers are trained using the softmax cross-entropy loss function as ruled in Equation (4), with $t_{i}$ being the truth label and $p_{i}$ being the softmax probability for the *i*th class that is set to only c=2 classes. Meanwhile, the weights and biases of the *n* FC layers are initialized randomly according to the normal distribution.

(4) $$L=-\sum_{i=1}^{c}t_{i}log\left(p_{i}\right)$$

#### 3.2.3. Tracker Model Update

**Figure 7:**
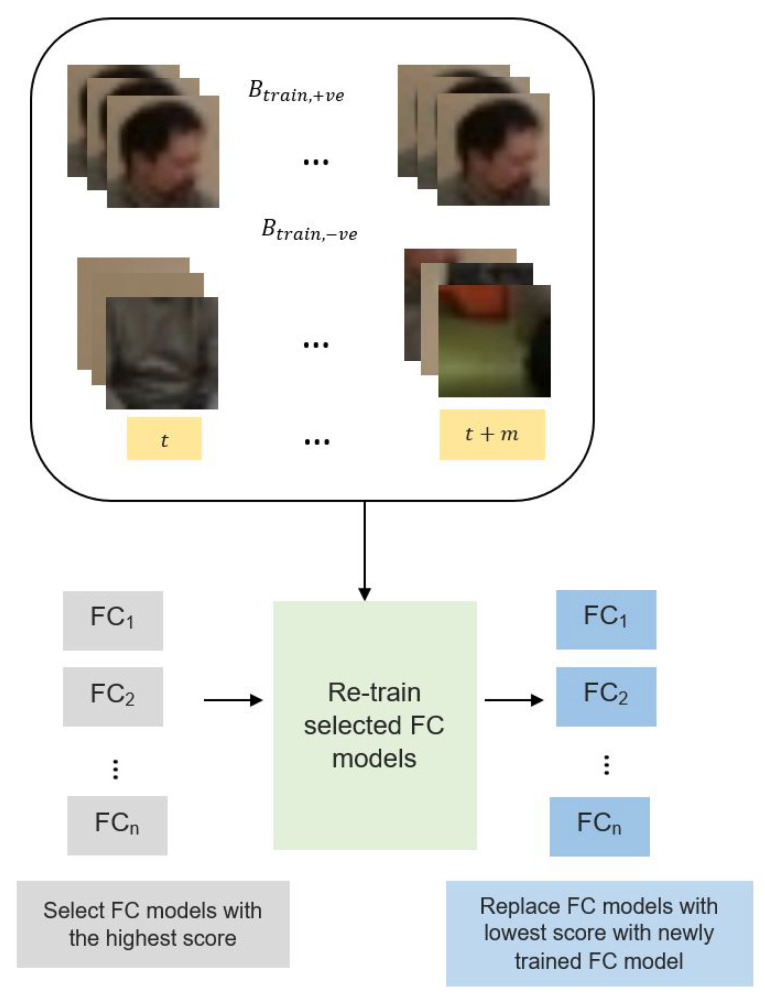
The full update process with *n* FC models.

The tracker needs to be updated periodically to circumvent model drift issues as the object appearance perceived from the camera will change when the tracked object moves from one frame to another. Figure 7 presents the full update process for our tracker with *n* FC models. A set of image patches, $B_{train,+ve}$ and $B_{train,-ve}$, are obtained by normal samplingaround $B^{t}$ with an intersection over the union limiting factor $F=\{f_{+ve},f_{-ve}\}$ set to 0.7 and 0.3, respectively. The implementation of *F* is performed to ensure both $B_{train,+ve}$ and $B_{train,-ve}$ are still sampled around the last updated location as it is a model-free tracker with no ground truth location given once the tracker has been initialized in the first frame. Moreover, the limiting factor is also imposed to consider size variations during the tracking process. Moreover, sampling background regions that are closer to the foreground have a consequential relationship compared to the far-away background. Both $B_{train,+ve}$ and $B_{train,-ve}$ are collected for a period of *m* recent frames. The model update process involves the selection of the FC model among the *n* FC models with the highest scores, which will be retrained using the accumulated $B_{train,+ve}$ and $B_{train,-ve}$. Then, the FC models with the lowest scores are replaced with the newly trained FC models. In addition, the FC models will be trained with a set of parameters as summarized in Table 3 for 50 epochs using Adam optimizer and a learning rate of 0.01.

(5) $$\begin{array}{cc} B_{train,+ve}∼\; & N(\left(B_{x}^{t},B_{y}^{t}\right),\sigma_{+ve})\\ s.t.\; & \frac{b_{i,+ve}⋂B^{t}}{b_{i,+ve}⋃B^{t}}>f_{+ve},\;i=\left\{0,\ldots ,N_{+ve}\right\} \end{array}$$

(6) $$\begin{array}{cc} B_{train,-ve}∼\; & N(\left(B_{x}^{t},B_{y}^{t}\right),\sigma_{-ve})\\ s.t.\; & \frac{b_{i,-ve}⋂B^{t}}{b_{i,-ve}⋃B^{t}}<f_{-ve},\;i=\left\{0,\ldots ,N_{-ve}\right\} \end{array}$$

Basically, the handling of the FC layers of our tracker shares the same fundamentals similar to the TCNN and MDNET-N trackers. However, some remarkable improvements have been made. One of these improvements lies in the implementation of multiple models, denoted by *n* models for the FC layers. The implementation of *n* FC models is proposed to handle the changes in the object appearance model and the occlusion issue during the tracking process. The consideration of maintaining a set of *n* FC models allows the tracker to perform well both during and after occlusion. The appearance model has a higher possibility of being updated with noisy information during occlusion, which can cause model drifting in the event that only a single model is maintained. It is difficult for a single model tracker to find the best match if it has been continuously updated with the occluded appearance information. As such, maintaining a set of *n* FC models reduces the noisy updates and coherently improves the tracking performances.

The TCNN tracker implements a tree structure to manage the FC models by maintaining the parent node and deleting the oldest node once a new child node is produced. The tree structure also involves the periodically updated process by accumulating the $B_{train}$ for a fixed frame interval. This technique allows the FC models to be updated using the best-matched appearance model rather than the most recent one. Similarly, our proposed tracker also implements the tree structure as imposed in the TCNN architecture. However, the parent node is not retained and will be replaced by the new child node. This modification is proposed to avoid skew in weight distribution towards particular branch modes, given that the selection of FC models is dependent on the highest scores. Moreover, this modification is imposed to evade the newly trained FC models from using the noisy information if the parent node is retained in the tree structure. On the other hand, the MDNET-N tracker manages the FC models by training with a large bias towards the last FC model, which is divided into several domains which correspond to the most recent training samples. Each domain will be trained separately and will be classified either as the tracked object or background. However, the noticeable weakness of the MDNET-N can be seen through the utilization of the recent training data, which can affect the future update of the appearance model if the training data are contaminated by the noisy or occluded information.

### 3.3. Stage 2: Instantaneous Fall Frame Detection

**Figure 8:**
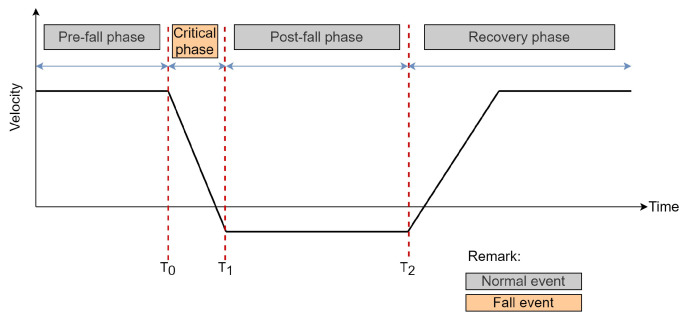
The main phases of a fall event.

In this subsection, the implementation of the instantaneous fall frame detection using the LSTM architecture is described. In recent studies, most of the existing fall detection systems are developed to classify the presence of fall events from typical events that occur in a video sequence. Contrarily, our goal is to detect the exact instantaneous fall frame. This specifically refers to the beginning of the ‘critical phase’ in which the fall event occurs, as illustrated in Figure 8. The detection of the instantaneous fall frame is crucial due to its close relationship with the response time that has become a critical issue in fall event detection. Moreover, the work in [74] pointed out that fall events occur instantaneously and rapidly within four seconds. Therefore, reducing the time between the occurrence of fall events and response time will allow a faster alert mechanism that will reduce the possibility of prolonged negative consequences such as injuries, fear of falling, and death. To the best of our knowledge, no existing fall detection system has been developed to specifically detect the instantaneous fall frame through the LSTM architecture. Henceforth, the LSTM model is employed to model the temporal information of the tracked object’s trajectories in discerning between the fall and no-fall features. The research conducted by [75] has corroborated that LSTM is an appropriate method to use in analyzing time sequence data.

#### 3.3.1. Training Module

**Figure 9:**
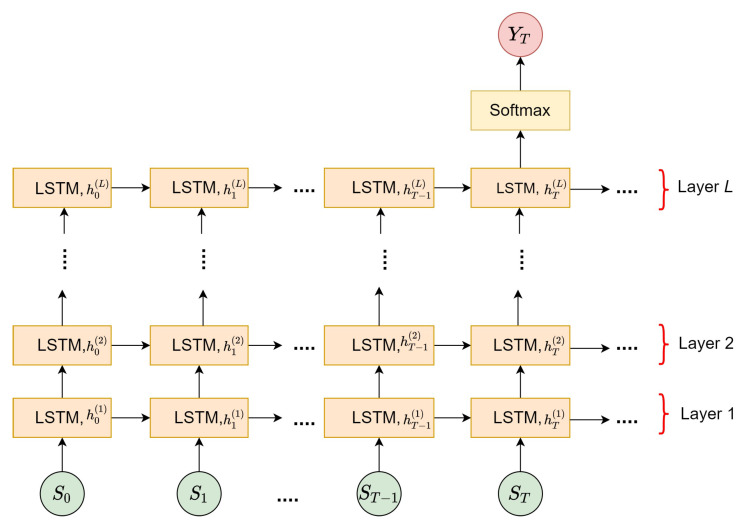
The proposed stacked LSTM model.

The proposed LSTM model is designed as a regression model which combines several LSTM layers on top of each other, as illustrated in Figure 9, with *L* representing the total number of layers with h=32 number of hidden states, $S_{t}$ denoting the input vector, and $Y_{T}$ representing the output score for every timestep, T=10. In addition, the stacked LSTM model is proposed due to its capability to extract more complex features over a single LSTM layer. Let *l* represent the current layer; the LSTM is used to relate each $S_{t}$ by mapping to its respective current $h_{t}^{l}$ and previous $h_{t-1}^{l}$ to produce the $Y_{T}$. Hence, at l=1, $h_{t}^{\left(1\right)}$ is obtained by mapping each $S_{t}$ to $h_{t-1}^{\left(1\right)}$ as ruled in Equation (7). From here on, the information of both $h_{t}^{\left(l-1\right)}$ and $h_{t-1}^{\left(l\right)}$ is used for the next subsequent layers. Finally, the $Y_{T}$ is produced by applying softmax cross-entropy loss function to the last $h_{T}^{L}$, which contains the probabilities distribution of two classes, c={fall, no-fall}. Each $h_{T}^{\left(l\right)}$ will be forwarded to the next T+1 to estimate the new output score, $Y_{T+1}$.

(7) $$h_{t}^{\left(1\right)}=\left(S_{t},h_{t-1}^{\left(1\right)}\right)$$

(8) $$h_{t}^{\left(l\right)}=\left(h_{t}^{\left(l-1\right)},h_{t-1}^{\left(l\right)}\right)$$

(9) $$Y_{T}=f_{L}\left(h_{t}^{\left(L\right)}\right)$$

**Figure 10:**
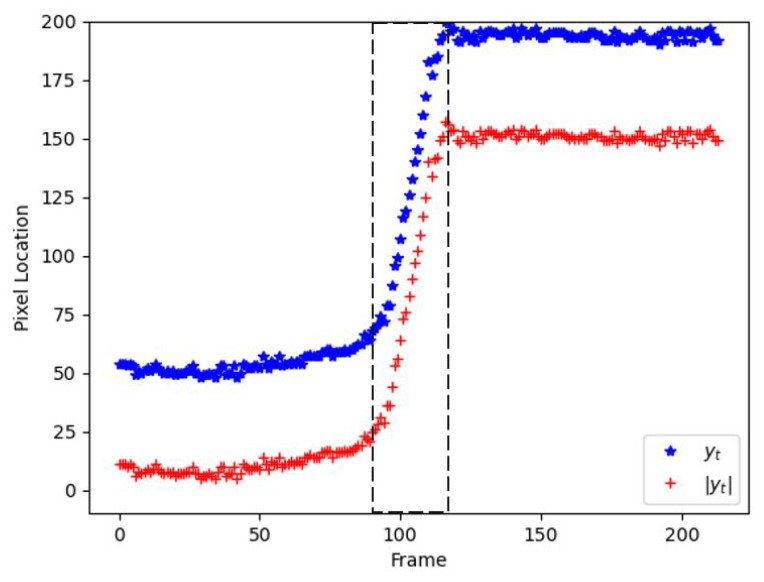
Comparison of pixel location for $|y_{t}|$ and $y_{t}$.

Four different training configurations are used to train the stacked LSTM model, as summarized in Table 4. These different training configurations are imposed to find the optimal training configuration to be used in detecting the instantaneous fall frame. The Train-1 configuration refers to the utilization of top-5 spatial object trajectories $\left(x_{t},y_{t}\right)$ for each $S_{t}$ that is obtained from the first module. Similarly, the Train-2 configuration adopts similar object trajectories, but with the additional dropout layers with a 0.5 dropout rate on the last stacked LSTM layer. These dropout layers are included with the intention to reduce the overfitting issue during the training process. Next, Train-3 configuration uses the combination of $x_{t}$ and normalized y-coordinate, $|y_{t}|$ for each $S_{t}$ in the training process. The $|y_{t}|$ refers to the differentiation values that relate each $y_{t}$ to the $y_{0}$ at the pixel coordinate (0,0) as ruled in Equation (10), with *V* denoting the total number of frames. Figure 10 displays the comparison between $|y_{t}|$ and $y_{t}$, in which the positions for $|y_{t}|$ are near the (0,0) coordinate, and vice versa for $y_{t}$. Moreover, the normalization technique is proposed to investigate the meaningful features, as $|y_{t}|$ indicates a large change during the critical phase in between t=97 and t=120, as in Figure 10. Meanwhile, the Train-4 configuration also employs a similar $\left(x_{t},|y_{t}|\right)$, but the implementation of dropout layers as in the Train-2 configuration. Furthermore, five-fold cross-validation is executed to test the performance of the stacked LSTM model, whereby each fold is tested independently with the training to testing data ratio being set to 4:1. The training procedures will be run for 1000 epochs with a learning rate of 0.000001 and a mini-batch size of 32, which is updated using an Adam optimizer. It is noteworthy to mention that these are optimal hyperparameters that have been selected using various hyperparameters testings.

(10) $$|y_{t,i}|=y_{i}-y_{0},\;i=\left\{1,\ldots ,V\right\}$$

#### 3.3.2. Decision-Making Module

**Figure 11:**
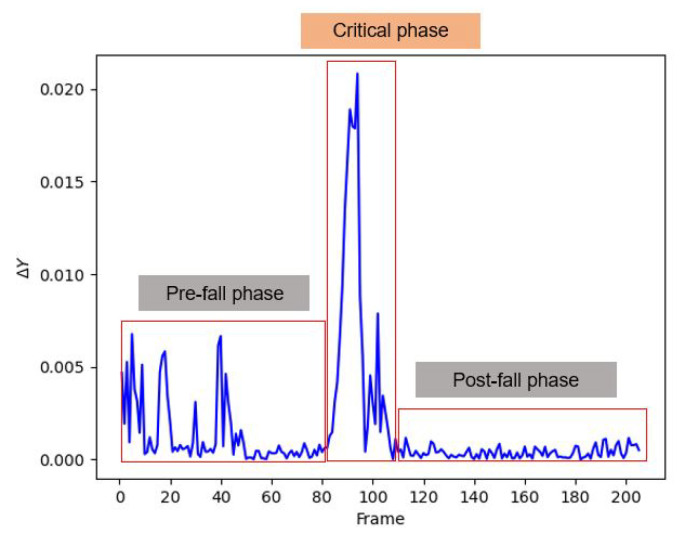
Comparison of $∆Y_{T}$ for three main phases of a fall event.

In this subsection, the decision-making module task is to locate the exact instantaneous fall frame using the stacked LSTM model that has been trained in the previous module. Letting $t_{exact}$ represent the exact instantaneous fall frame, $t_{exact}$ is determined based on the score differences of $Y_{T}$ at the current frame from the previous frame as defined in Equation (11), with $∆Y_{T}^{i}$ denoting the score differences, *i* being the current frame, i−1 representing the previous frame, *V* being the total number of frames, and *T* being the timestep. Then, the $t_{exact}$ is located by finding the index frame of the highest $∆Y_{T}^{i}$. Finding the highest $∆Y_{T}^{i}$ is imposed due to the large changes in $∆Y_{T}$, as illustrated in Figure 11, which coherently indicates the presence of the fall frame. Moreover, the figure also displays the comparison of $∆Y_{T}$ for three phases of a fall event, where $∆Y_{T}$ has the biggest changes from the pre-fall period to the critical phase, which supports the occurrence of the fall frame. Please note that no recovery phase is presented in the FDD dataset since all the actors remain lying on the floor after the fall event simulations.

(11) $$∆Y_{T}^{i}=\left|Y_{T}^{i}-Y_{T}^{i-1}\right|,\;i=\left\{1,\cdots ,V-T\right\}$$

(12) $$∆Y_{highest}^{i}=max\left(|∆Y_{T}^{i}|\right)$$

(13) $$t_{exact}=argmax\left(∆Y_{highest}^{i}\right)+T$$

### 3.4. Performance Evaluation

The performance evaluation of the object tracking module will be measured using four Visual Object Tracking (VOT) Challenge [76] protocols, comprising of accuracy (Ac), robustness (Ro), reliability (Re), and expected area overlap (EAO). These performance metrics are de facto metrics used as the benchmark to measure the performance of the proposed tracker. Note that the EAO is the primary measure used to rank the tracker performances, as used in the VOT Challenge. Yet, the performance observations on Ac, Ro, and Re are necessary; as such, the tracker model training and model update are dependent on the implementation of hyperparameters that are closely related to each other. Ac measures the average intersection over union (IoU) areas between the tracker output bounding box, $B_{output}$, and the ground truth bounding box, $B_{gt}$, in a video sequence of *V* length, as defined in Equation (14), with *S* representing the total number of video simulations. However, Ro measures the average of tracking failures, $F_{fail}$, and as such $F_{fail}$ is triggered when there is no IoU between $B_{output}$ and $B_{gt}$. Re captures the likelihood of successful tracking after *Q* frames that have been fixed to 100 frames. EAO measures the average IoU over a range of frames between [lim^low^, lim^up^]. EAO is imposed to rank the tracker by considering the trade-off performance between Ac and Ro without adopting any re-initialization protocol. EAO is measured along the frame of *V* length before the first $F_{fail}$ is triggered; as such, Ac values on each frame thereafter will be ignored. Moreover, each tracking process is repeated 15 times as set by the VOT protocol to obtain a good stochastic measurement. Meanwhile, the mean error is used to evaluate the performance of the instantaneous fall frame module, in which it measures the average error between the $t_{exact}$ and the provided ground truth, $t_{gt}$.

(14) $$Ac=\frac{1}{S}\sum_{i=1}^{V}\frac{B_{output}^{i}⋂B_{gt}^{i}}{B_{outout}^{i}⋃B_{gt}^{i}}$$

(15) $$Ro=\frac{1}{S}\sum_{i=1}^{V}F_{fail}^{i},\;F_{fail}^{i}=\left\{\begin{array}{cc} 1, & \mathrm{if}\;IoU_{i}\leq 0\\ 0, & \mathrm{if}\;IoU_{i}>0 \end{array}\right.$$

(16) $$Re=\frac{e^{-Q\frac{Ro}{V}}}{S}$$

(17) $$EAO=\frac{1}{S}\left[\frac{1}{lim^{atas}-lim^{bawah}}\sum_{i=lim^{low}}^{lim^{up}}IoU_{i}\right]$$

(18) $$Mean\;error=\left|t_{exact}-t_{gt}\right|$$

## 4. Results

This section firstly describes the individual results for each training configuration of the object tracking module for the optimal setup of the SmartConvFall tracker. In addition, the proposed SmartConvFall will be benchmarked with state-of-the-art deep learning-based trackers, namely TCNN and MDNET-N, that share similar tracking fundamentals. This section also discusses the final results of the exact instantaneous fall frame detection performance.

### 4.1. Results: Stage 1—Object Tracking Module

**Figure 12:**
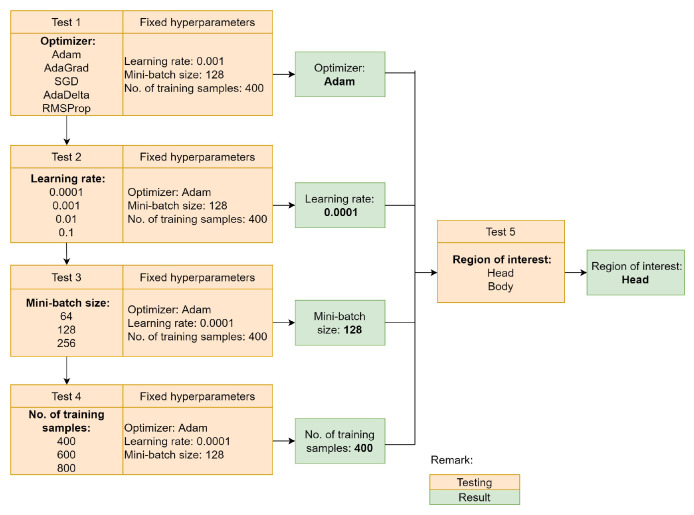
The full training configuration results for each tested hyperparameter.

The tracker training phase in the first frame is crucial to produce a good model-free tracker, whereby a slight error can affect the model performance of the entire tracking process. Therefore, this section discusses the results of the optimal training configurations for our proposed SmartConvFall tracker. Figure 12 presents the full training configuration results that have been tested individually in the first frame to obtain the best-trained tracker model. The discussion of each test is provided separately in the next subsections.

#### 4.1.1. Optimizer

The first test examines the impact of the different optimizers, namely Adam, AdaGrad, SGD, AdaDelta, and RMSProp, with a fixed learning rate, mini batch size, and the number of training samples set to 0.001, 128, and 400, respectively. Table 5 compares the evaluation metrics for each optimizer of both regions of interest. The experimental results show that the Adam optimizer achieves the highest EAO with 0.1668, followed by RMSProp, AdaGrad, SGD, and AdaDelta for the head region. Meanwhile, RMSProp achieves the highest EAO, with 0.1708 for the body region. The highest EAO reveals the tracker’s capability to perform the tracking process by emphasizing the performance of both Ac and Ro. As seen from Table 5, the Adam optimizer tested with the head region produces the top results for Ro, Re, and $F_{fail}$ with 0.4493, 0.8557, and 31, respectively. Despite having the second-best Ac with 0.4415, the Adam optimizer still manages to produce the lowest $F_{fail}$ when compared to the RMSProp. A low $F_{fail}$ indicates that the tracker experiences few tracking failures, whereas a high $F_{fail}$ reveals that the tracker needs to undergo a repeated re-initialization protocol. In this process, the $B_{gt}$ will be re-supplied, directly causing the increasing values of Ac in the early stage after the re-initialization process. Similarly, the Adam optimizer produces the top results for the Ro, Re, and $F_{fail}$ when tested with the body region of interest.

The good performance results of the Adam optimizer explain its capability to update the network parameters while reducing the loss function to zero. Moreover, the Adam optimizer employs an adaptive learning rate protocol for each involved parameter during the model updating process. In fact, the algorithm behind the Adam optimizer combines the uniqueness of the RMSProp and AdaGrad to produce a better optimization technique that can rapidly converge to the optimal settings. Similarly, the RMSProp optimizer also utilizes an adaptive learning rate protocol, yet the learning rate decay is based on the average gradient, which is slightly different from the Adam optimizer that uses previous average gradient information. The adaptive learning rate is also implemented in the algorithm of the AdaGrad optimizer; however, the weight update process causes the learning rate value to continually decrease, which affects the model performance. Meanwhile, the AdaDelta optimizer is an improved version of the AdaGrad, still using a rapid decayed learning rate, which hinders the model from learning the features efficiently. On the other hand, the SGD optimizer requires a longer convergence time, which explains its low performance as compared to the Adam optimizer. Therefore, the Adam optimizer is chosen to be the best optimizer for the SmartConvFall tracker based on the top results of EAO, Ro, and $F_{fail}$ for both regions of interest.

#### 4.1.2. Learning Rate

**Figure 13:**
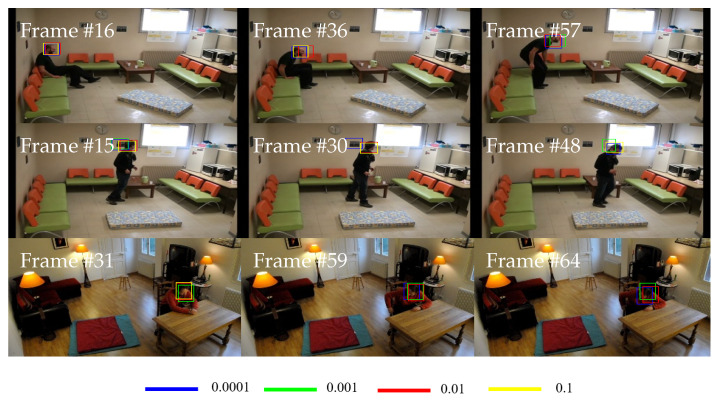
Some image samples of $B_{output}$ for all learning rates, showing $F_{fail}$ in the beginning of tracking.

Table 6 lists the overall results for the learning rate configurations set to 0.0001, 0.001, 0.01, and 0.1. For a fair comparison, each testing is evaluated individually with fixed hyperparameters using the Adam optimizer, a mini-batch size of 128, and 400 training samples. It can be seen from Table 6 that a learning rate of 0.01 yields the highest EAO with 0.1693 for the tracking performance using the head region of interest. Contrarily, the highest EAO is produced by a learning rate of 0.0001 with 0.1898 for the body-based region of interest. The highest EAO is yielded by the learning rate of 0.0001 coming from the body-based region of interest, and this is also supported by the best results produced by Ac, Ro, Re, and $F_{fail}$. Nevertheless, the learning rate of 0.0001 for the head-based region of interest only manages to produce the second-best EAO. This is due to the protocol of EAO that is dependent on both Ac and $F_{fail}$. The tracking failure at the beginning of tracking will affect the overall performance, as compared to the tracking failure that occurs towards the end of the video sequence. Figure 13 depicts some samples $B_{out}$ showing a $F_{fai}$ incident that occurs at the beginning of the tracking process.

These results explain that an optimal learning rate value plays an indispensable role in producing the best tracker because it controls the step size used in updating the model weights. Moreover, the obtained results also demonstrate that finding the best learning rate is difficult to achieve due to the learning rate decay process that also depends on the optimization technique. Nevertheless, it can be observed that using a high learning rate such as 0.1 produces low-performance results since the tracker is more likely to learn more on the background information. Contrarily, a small learning rate indicates that a small step size is used to update the model weights that cause the tracker to learn more on the tracked object information. Therefore, the learning rate of 0.0001 is selected as the best learning rate to be employed in the tracker model training.

#### 4.1.3. Mini-Batch Size

This test is performed to scrutinize the impact of different mini-batch sizes on the tracker model training. Table 7 presents the performance evaluations of the mini-batch size configurations. The best set of optimal hyperparameters that were obtained from the previous tests are the Adam optimizer and the learning rate of 0.0001, which are also used as the basic configurations for this test. From Table 8, it is apparent that the highest EAO is produced by a mini-batch size of 64 for both regions of interest, with 0.1679 and 0.1683, respectively. Specifically, a mini-batch size of 64 for the head region manages to obtain a higher Re even though it has higher $F_{fail}$ compared to the body-based region of interest. Interestingly, a mini-batch size of 128 for both regions of interest has similar $F_{fail}$ values. However, the tracker’s ability to track the object is better when using the configuration of the head-based region of interest that produces Re of 0.8557 compared to the body-based region of interest. Similarly, a mini-batch size of 128 and 256 have similar $F_{fail}$ and Ro values for the body-based region of interest. Despite this fact, the tracker’s ability through Re for a mini-batch size of 256 is lower than the mini-batch of size 128, despite similar $F_{fail}$ results, with a total of 31 failure cases.

This experiment corroborates the findings for the optimal selection of mini-batch size, which can eventually affect the tracker model performance. Mini-batch size is introduced during the training process to control the size or the number of training samples for each backpropagation update, whereby the deep learning model can be optimally trained by using a subset of the training data. If the full dataset is used for each parameter update, the weight will not learn much as the model will be globally generalized, whilst, if a small batch size is used, the parameter update will experience extreme fluctuation in the case of noisy data. Furthermore, the selection of mini-batch size is also constrained by the available computer and graphics processing unit memories; as such, a mini-batch size of 256 is the upper limit for this experiment. Additionally, the results for this experiment are also greatly dependent on the distribution of positive and negative training samples for each batch, which will be randomly queued. Note that EAO is the most important evaluation metric used to rank the tracker performances, yet the performance observations of Ro, Re, and $F_{fail}$ should also carry some weight to avoid some extreme cases. Moreover, the training of the deep learning tracker requires the implementation of hyperparameters that are closely related to each other, such as optimization method and learning rate values. Henceforth, the mini-batch size of 128 is selected as the best training configuration for the proposed SmartConvFall tracker. Although it only manages to produce the second-best and third-best EAO for the head and body-based regions of interest, respectively, these values are still acceptable without much fluctuation in other performance metrics. The selection of a mini-batch size of 128 is also supported by the significant results of Ro, Re, and $F_{fail}$ for both head-based and body-based region of interests.

#### 4.1.4. No. of Training Samples

This subsection investigates the best combination of training sample densities to be used in training the tracker. A dense sampling will result in many repeated samples which will increase the computational burden, while a low sampling number will increase the likelihood of missed detection due to the uneven bounding boxes generation. Each test is conducted using similar settings with an Adam optimizer-based update, a fixed learning rate of 0.0001, and a mini-batch size of 128 for a fair comparison. Table 8 provides the full evaluation results for all three configurations of the training samples. Letting $B_{train}$ represent the number of training samples, $B_{train}=400$ produces the highest EAO of 0.1669, followed by $B_{train}=600$ and $B_{train}=800$ for the head-based region of interest. Meanwhile, $B_{train}=600$ yields the highest EAO when tested using the body-based region of interest. Moreover, it can be observed that $B_{train}=400$ for the head-based region of interest manages to achieve the highest Re of 0.8557. In addition, $B_{train}=400$ also produces the smallest $F_{fail}$ and Ro. Despite having the highest Ac for $B_{train}=600$ for both regions of interest, this training sample density also produced an increased number of $F_{fail}$ cases with 36 and 32 for the head- and body-based regions of interest, respectively. Interestingly, $B_{train}=600$ and $B_{train}=800$ for the head-based region of interest produces similar results for Ro and $F_{fail}$. Nonetheless, $B_{train}=600$ captures the best tracking likelihood of every 100 frames.

**Figure 14:**
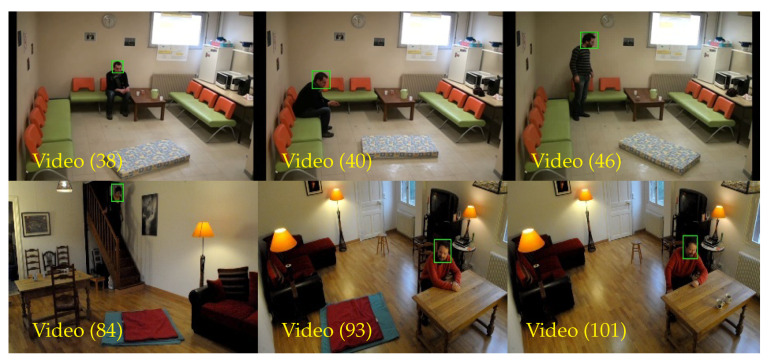
Image samples that showing tracked objects having similar information with the background information.

The training process for a deep learning-based technique requires a large number of training samples due to the usage of complex network architecture as compared to the standard machine learning techniques. However, the main constraint of deploying a deep learning model for tracking applications is limited variations in the training data, whereby only first-frame information is used to train a model-free tracker. Therefore, the transfer learning method is adopted to overcome the limited number of training samples in this work. Through a transfer learning method, pre-trained weights and biases of the VGG-M model are used during the initialization phase. The VGG-M model has been trained using the ImageNet database [77] which consists of more than one million images with 1000 classes. Additionally, the VGG-M model is built as a general classification model without specification towards any application. Yet, these pre-trained weights and biases can be used in object tracking applications, specifically for the fall detection domain, by training the proposed CNN networks with the specific training samples that are not too large. Moreover, there are cases in the FDD dataset in which the person to track is far from the camera’s point of view, and as such the bounding boxes that encapsulate the region of interest are of a smaller size, especially for the experiment that uses a head-based region of interest where the lowest width of the bounding box is just 18 pixels. Coherently, only a small number of sampling candidates can be obtained due to the small region of interest. One way to handle the limited training samples is by the utilization of the translation strategy that is imposed in this work that fixed the translation stride for a maximum of 10 pixels to ensure that the sampling candidates still manages to extract about 40% of the ground truth information. This is set so that the tracker still manages to learn the objectness information of the tracked object even though the region of interest is very small. Henceforth, finding the appropriate number of training samples is crucial so that the tracker can properly learn the features of the tracked object. Thus, $B_{train}=400$ is selected as the best number of training samples to train the tracker model in the first frame. Moreover, $B_{train}=600$ is chosen based on the significance results of Ro, Re, and $F_{fail}$ for both regions of interest. Through this experiment, a large number of training samples, such as $B_{train}=800$, will raise the possibility of overfitting issues to the SmartConvFall tracker due to the frequent repeated similar samples, whereby a lot of background information is also introduced as depicted in Figure 14. It contains bounding box samples from Videos (38), (40), (46), and (84) where the object appearance (hair color) is almost similar to the background (clothes color). Meanwhile, Video (93) and (101) depict situations where hair color is relatively similar to the furniture color that is located behind the actor. Hence, these situations make it harder for the tracker to distinguish the tracked object from the background, which consequently influences the tracker’s performance.

#### 4.1.5. Region of Interest Selection

**Figure 15:**
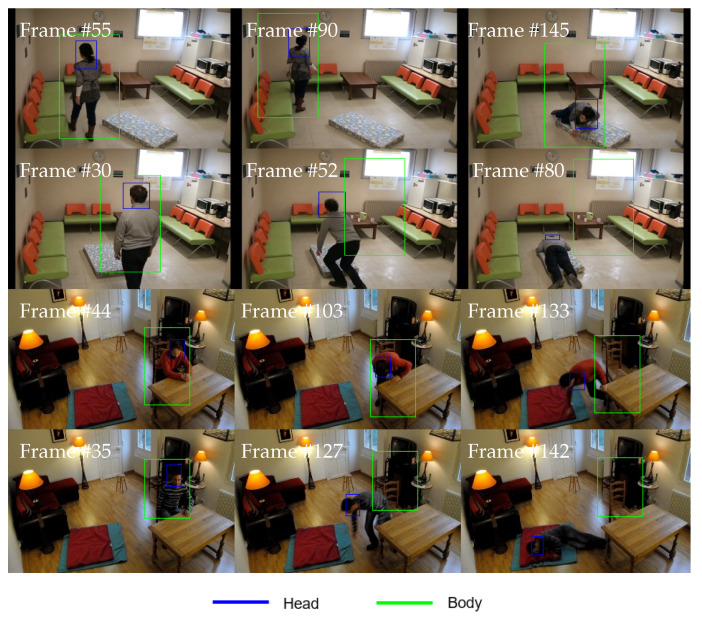
Comparison of $B_{output}$ for both head and body regions of interest.

In this subsection, the selection of the best region of interest to be tracked by the SmartConvFall tracker is analyzed for optimal appearance representation. The previously determined optimal hyperparameters are the Adam optimizer backpropagation method, a learning rate of 0.0001, a mini-batch size of 128, and 400 training samples being employed to select between head- or body-based regions of interest. The full performance results for all tests are shown in Table 9. It is apparent from the results that tracking only the head region produces better performance results compared to tracking the upper body region, with EAO, Ac, Ro, Re, and $F_{fail}$ of 0.1718, 0.4871, 0.4706, 0.8274, and 32, respectively. Moreover, these results also indicate that the proposed CNN networks can learn and extract significant features from the head region, even though the size is smaller. Henceforth, the head region is selected to be the tracked region of interest for the entire tracking process. Figure 15 displays some output samples $B_{output}$ of the tracker using both regions of interest.

### 4.2. Results: Stage 2—Instantaneous Fall Frame Detection Module

This subsection discusses the simulation results for the exact fall frame ($t_{exact}$) detection using the proposed stacked LSTM network that takes input from the optimized SmartConvFall tracker. The existing related works on fall detection systems utilizing the LSTM architecture are based on the following classification approach: fall features are classified from non-fall features with no concern in regard to when the fall event occurred. However, our works propose a different technique, in which we aim to determine the fall frame, denoted as $t_{exact}$ immediately for each input sequence that is supplied to the stacked LSTM architecture. The proposed stacked LSTM architecture is treated as a regressor instead of a classifier to determine the $t_{exact}$. The aim of this approach is to reduce the negative consequences of delayed treatment. For example, if a segment of a 15 s video is used to detect the fall event, the notification is only triggered after the 15 s. This is because the whole sequence of the video is needed to make the decision when the classification approach is used. However, due to the non-availability of the related works that work on a similar approach, we could not execute a performance comparison with our proposed approach. Henceforth, the proposed stacked LSTM network is compared to the RNN model; both models belong to the similar deep learning class that comprehends information of the time-series data. The basic strategy used to identify $t_{exact}$ is analyzing the highest score differences, $∆Y_{highest}$, for each subsequent frame. The average mean error metric is used to evaluate the procedure, which is obtained by finding the average difference between $t_{exact}$ and its ground truth, $t_{gt}$. Moreover, the performance of the stacked LSTM layer is benchmarked with RNN, as no RNN-based technique has been designed based on the tracker output to detect the exact fall frame. The mean error results are shown in Table 10 for both stacked LSTM and RNN models. Based on the table, the average mean error using both F1 and F2 configurations for RNN are 40 and 43 frames. Meanwhile, the average mean error for F3 and F4 configurations are 40 and 43 frames, respectively. It is worth noting that LSTM with a single layer, L=1, produces the lowest mean error of 32 frames using the F1 configuration, followed by F4 configuration with a mean error of 33 frames. There is only a slight performance difference with just one frame error between the mean error of F2 and F3 configurations. In conclusion, a single-layer LSTM achieves better detection results and proves the effectiveness of the LSTM network over the RNN in modeling the tracker’s temporal relationship.

**Figure 16:**
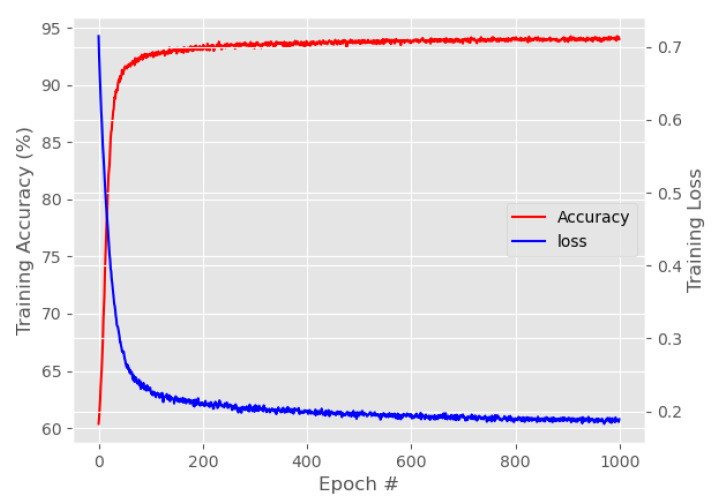
Graph of training accuracy and loss for stacked LSTM with L=3.

Apart from comparing the performance of a single layer of LSTM and RNN, this subsection experiments on various layer configurations of a stacked LSTM network in finding the $t_{exact}$. The basis of F1, F2, F3, and F4 configurations still remains the same, where a basic F1 configuration delivers good detection performance of $t_{exact}$ with a mean error of 29 frames. Nevertheless, a further increment in the LSTM stacking using the F1 configuration does increase the mean error, whereby the highest mean error is produced by L=7 with 44 frames. However, by using the F2 configuration, stacked LSTM achieves the lowest mean error of 27 frames for both L=3 and L=4. Similarly, the F2 configuration shows an increasing mean error trend once the stacked layers exceed L=4. A similar situation is observed for the F3 configuration with an increasing mean error starting from L=3 until L=7. Surprisingly, the F4 configuration produces the lowest mean error of 22 frames when L=3 is used. However, a similar pattern is observed when further experiments were performed using L>3. Overall, a three-stacked LSTM using F4 configuration produces the best performances and it will be used in this work to detect $t_{exact}$. Moreover, further evaluations in terms of training accuracy and loss were also evaluated to corroborate the selection of F4 configuration, as shown in Table 11. It is apparent from Table 11 that L=3 produces the top results with 93.1% training accuracy and 0.2112 loss as illustrated in Figure 16. On the other hand, RNN produces the lowest detection performance with just 59.17% training accuracy and the corresponding high training loss of 3.1578. Moreover, the results also reveal that an increasing trend of training accuracy for stacked LSTM can be observed, starting from L=1 until L=3, and then, it begins to decrease from L=4 until L=7. This observation is in line with the findings from the mean error results, as shown in Table 10. Furthermore, a stacked LSTM with big *L* requires a longer training duration due to the increased model complexity in learning the provided temporal information.

Therefore, it can be concluded that the F4 configuration, which utilized the information of $|y_{t}|$ by relating each y-coordinate, $y_{t}$, to the $y_{0}$ at the pixel location (0,0), produces the most accurate detection scheme in locating the $t_{exact}$, contrarily to the F1 and F2 configurations that only relate the information of $y_{t}$ without further normalizing them, with the observation of $|y_{t}|$ limiting the detection network in learning their temporal relationship, which manifests through poor performances in locating the $t_{exact}$. Additionally, the F4 configuration also utilizes a dropout layer, which reduces the overfitting possibility by randomly dropping some of the node’s connections. Henceforth, it can be concluded that a stacked LSTM of three layers using the F4 configuration is the optimal setting for locating the $t_{exact}$. Moreover, this configuration manages to produce the lowest mean error of just 22 frames, which translates to a less than 1 s response time if the input video is streamed with a 30 fps format. Additionally, the proposed stacked LSTM model produces better performances compared to the RNN due to its better hierarchical arrangements of the hidden state, which enables the network to capture more complex temporal representations at different scales. Lastly, the obtained results fulfil the main objective of this paper in designing an exact fall frame detection system, which is critical for immediate response time to reduce the negative consequences of a fall incident.

### 4.3. Benchmark Comparison

**Figure 17:**
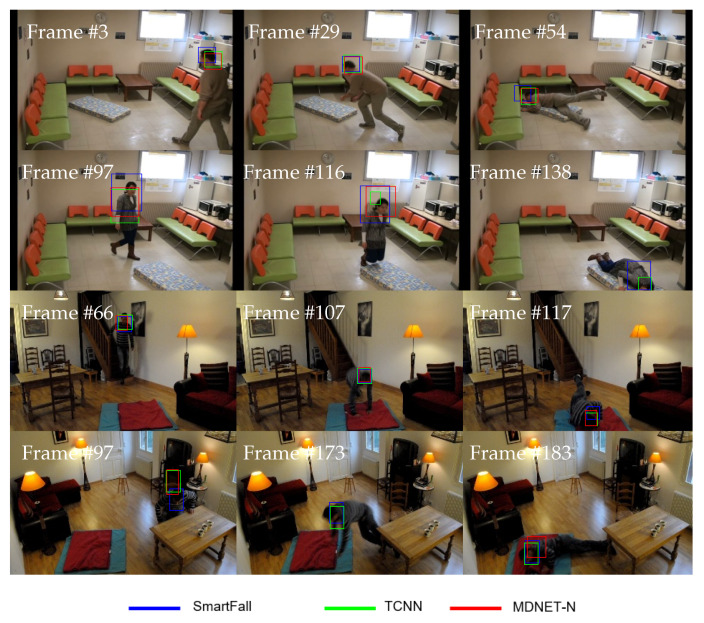
Comparison of $B_{output}$ of all tested trackers for no-attribute videos.

This section discusses the SmartConvFall tracker performances compared to the state-of-the-art CNN trackers, namely TCNN and MDNET-N trackers. Both TCNN and MDNET-N incorporated a fully CNN-based network as the core tracking mechanism. This mechanism relates the same object between consecutive images. These trackers essentially share similar tracking fundamentals, particularly in regard to the design of convolutional layers. However, they differ in the way of handling and updating the FC layers to differentiate the tracked objects from the background information. For a fair comparison, the optimal settings for the TCNN and MDNET-N trackers have been chosen and selected based on the suggested optimal setup in the original papers. We have also confirmed that both of the models have been trained to convergence. These trackers are initially evaluated using a set of videos from the FDD dataset; the set contains minimal visual noise attributes. Each tracker is subsequently tested by considering two challenging noise attributes, which are occlusion and a cluttered background to mimic real-life usage scenarios. Table 12 presents the performance results of each tracker for the noise-free test videos. The SmartConvFall tracker performs the best with the highest EAO of 0.1619, followed by MDNET-N and TCNN with 0.1525 and 0.1436, respectively. Despite having the lowest Ac, SmartConvFall is relatively more reliable, with the best Re of 0.7958. Moreover, SmartConvFall is able to track the object with the lowest number of $F_{fail}$ cases; only 43 tracking failures were found throughout the entire set of video sequences. Even though MDNET-N and TCNN produce better Ac, this improvement can be attributed to the frequent re-initialization process; both TCNN and MDNET-N reported almost twice the number of $F_{fail}$ cases compared to the SmartConvFall tracker. This frequent re-initialization impact can be seen through a lower EAO, whereby the Ac is not considered after the first $F_{fail}$ case. It must be noted that an $F_{fail}$ case happens when there are no overlapping areas between the $B_{ouput}$ and $B_{gt}$. Hence, $B_{gt}$ information is re-supplied for a model-free tracker initialization, which then leads to better $B_{ouput}$ right after the re-initialization process. Figure 17 depicts some $B_{output}$ for SmartConvFall and its benchmarked trackers.

**Figure 18:**
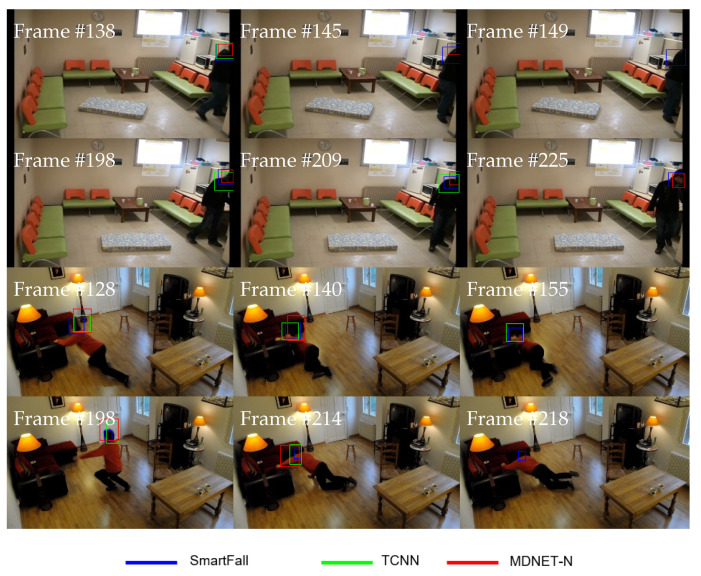
Comparison of $B_{output}$ of all tested trackers for occlusion noise attribute.

Table 13 lists the tracking results for all three trackers tested on video sets with occlusion attributes. Occlusion is one of the challenging issues in visual object tracking, whereby the tracked object can be either fully or partially occluded from a camera’s point of view. Occlusion noise greatest affects a tracker that is designed based on a track-by-detection philosophy as it relies more on appearance information compared to the spatial relationship. Again, the SmartConvFall tracker achieves the best results of EAO, Ro, Re, and $F_{fail}$ with 0.1713, 0.7501, 1.0833, and 13, respectively. Although the SmartConvFall tracker employs a tree structure in managing the FC nodes, similar to TCNN, its parent node is deleted once the child node is created to avoid redundancy in the model pool, as well as limiting the contribution of an occluded node. Moreover, the obtained results also corroborate the advantage of maintaining multiple models for the FC layers in resolving the occlusion issue. The disadvantage of a simple update mechanism as used in TCNN and MDNET-N leads to a higher possibility of model drift due to the noisy update. Table 13 also reveals that the TCNN tracker obtains a higher EAO compared to the MDNET-N tracker, which indicates that maintaining a set of FC nodes in a tree structure produces better performance, instead of updating the recent node only. Moreover, the update protocol used in MDNET-N that relies heavily on recent training samples causes the tracker to be updated with noisy information, which directly affects the tracker’s performance. Additionally, SmartConvFall is also able to track the object of interest with the lowest number of tracking failures, which proves its ability in handling occlusion issues. Figure 18 illustrates some $B_{ouput}$ comparisons of all tested trackers tested on videos with occlusion attributes.

**Figure 19:**
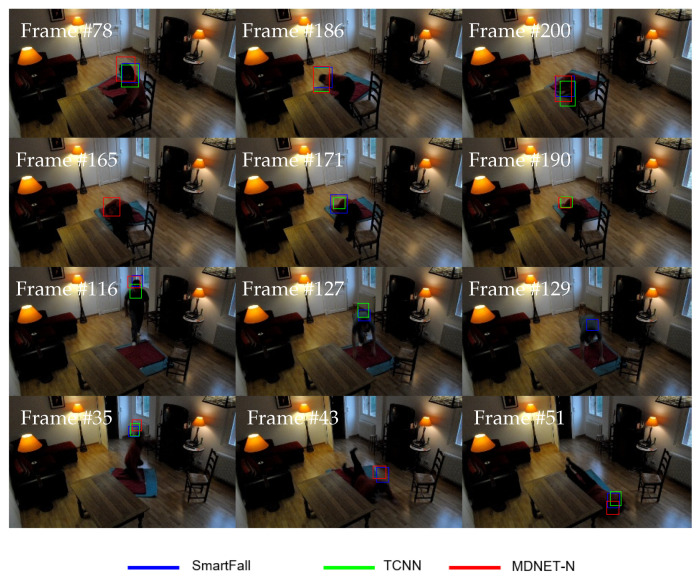
Comparison of $B_{output}$ of all tested trackers for the cluttered background attribute.

Meanwhile, Table 14 presents the performance results for the trackers tested on videos with a cluttered background attribute. A cluttered background is a condition in which the nearby objects have close appearance resemblance information to the tracked object, as depicted in Figure 19 with its respective $B_{output}$. The obtained results show that SmartConvFall is the top tracker with the highest EAO of 0.1509, followed by MDNET-N and TCNN trackers. Interestingly, SmartConvFall also manages to achieve the top results for all performance metrics, Ac, Ro, Re, and $F_{fail}$. The good capability of the SmartConvFall tracker in overcoming the cluttered background noise attribute can be traced back to the usage of the effective multiple model of FC nodes. Meanwhile, TCNN protocol that is emphasized more in the recent model in its update procedure causes the model to be updated with contaminated noisy information. Similarly, the MDNET-N tracker updates its FC nodes based on the latest training samples only, sampled at a fixed frame interval, which leads to having a lower FC score when encountering a cluttered background situation. As a consequence, the tracker’s ability for the subsequent frames will be affected. Moreover, Table 14 also indicates that both TCNN and MDNET-N trackers yield similar performance for Ro, Re, and $F_{fail}$ metrics. Nonetheless, the TCNN tracker encounters a tracking failure case earlier than the MDNET-N tracker, which results in a lower EAO and higher Ac due to a more frequent re-initialization protocol being executed. Therefore, the experimental results have validated the SmartConvFall capabilities by maintaining multiple models of FC nodes to overcome any occluded and cluttered background noise.

## 5. Discussion

This paper proposes SmartConvFall for fall detection based on the instantaneous fall frame using two subsequent deep learning models: CNN and LSTM. Our work aims to identify the exact fall frame instantaneously through a regression network rather than to classify a video clip whether it contains a fall event or not. Hence, the performance metric used in this work is the mean error that quantifies the mean differences between the detected frame and its ground truth, instead of the standard performance measure of the classification task, such as recall and precision. Moreover, we have also implemented tracking outputs, which are the movement trajectories as the inputs to the regression network. Therefore, several state-of-the-art CNN-based trackers are used as the performance benchmark. The works in [57,59,78] have adopted a compact CNN design in their proposed systems with two, three, and five convolutional layers, respectively. Similarly, we have proposed a compact CNN architecture by utilizing only three convolutional layers. The implementation of the compact CNN architecture is essential to reduce the under-fitting issue [79] that is caused by limited training samples in updating the tracked object model. Furthermore, the compact design allows for real-time implementation and it is more suitable for mobile platform development. On the other hand, a deeper CNN architecture requires a longer training time due to the increasing number of convolutional layers, as implemented in [58,61,62]. The increasing number of convolutional layers increases the computational complexity of the systems. Hence, a fast response time cannot be produced for fall detection systems that depend on deep CNN architecture.

The nature of fall events that infrequently happen in real-life scenarios has contributed to another challenge for the vision-based fall detection system. Real-life fall events are scarce and difficult to acquire. However, most of the previous works make an ideal assumption on the training samples. Most of these works have used self-collected fall events videos under a controlled setting, which might pose a different challenge than the actual fall simulations. This will create a situation whereby the fall event can be easily triggered, as most of the posed action is not performed accurately. For example, Han et al. [64] have included twice the number of falling actions in the training phase rather than the walking poses. Conversely, Min et al. [61] have introduced a set of more typical human poses such as walking, sitting in a chair, sitting on a sofa, and lying on a sofa. Hence, this situation can affect the fall detection results. Likewise, our work uses simulated falls obtained from the FDD dataset. However, the biggest concern regarding the distribution of the fall events from the typical human poses has been considered. We have excluded these ADL video sequences from the experiment. This is to ensure that the CNN network is not trained in a biased manner towards typical human poses which can influence our system’s performance.

Occlusion and background clutter issues are also critical in vision-based fall detection systems. For instance, a home-like environment often contains many surrounding objects, such as a wardrobe, sofas, and tables. These objects may cause occlusion issues when a person walks behind one of these items. In addition, the object’s appearance may also look too similar to the person of interest, especially the color. These issues can deteriorate the performance of the fall event detection system. Therefore, head tracking/segmentation has been adopted in vision-based fall detection systems. Our work has used the head information as the region of interest to the tracking network so that the challenges by occlusion and background clutter can be minimized. Similarly, Yao et al. [51] and Adhikari et al. [80] have also employed head tracking in their fall detection system. However, there is also an existing work that does not address these issues specifically, such as the work by Li et al. [59], whereby their system performance is not very accurate.

At the same time, we have taken a step further in minimizing these crucial issues. Our study aims to determine the fall frame immediately for each input sequence, which is in contrast to the recent fall detection approaches that only decide the occurrence of a fall event after a period of time. The fall frame detection for our SmartConvFall is achieved through the integration of a stacked LSTM model. Moreover, Mobsite et al. [22] have also employed the LSTM model to provide a time-based fall classification. However, the proposed technique is not suitable for online implementation because they have modified the training data by deleting the first and last frames of the fall videos. This interference can influence the response rate and does not provide the solution to reducing the negative fall consequences. Similarly, the work in [23] has adopted the LSTM model for the fall classification approach using the key-points representation of the human poses. Nevertheless, the proposed system is developed for multiple-person surveillance applications that are not in our work’s scope. We have put emphasis on single-person monitoring that targets the elderly or people who live alone at home. We believe that if more than two persons are available at a scene, the fall detection system is not needed as the other person can always notify the caretaker immediately in the case of any fall event occurring.

## 6. Conclusions

This paper presented extensive simulation tests of various network configurations for the proposed SmartConvFall tracker in detecting the exact instantaneous fall frame. The SmartConvFall tracker relies on deep learning representation. This entails a fully CNN-based tracker that is employed to track the object of interest in a video sequence. Moreover, the transfer learning approach was also adopted to improve training accuracy due to limited training data in a model-free tracker. Hence, optimal training configurations are crucial to ensure optimized training settings for the network parameter update. Moreover, we integrated a stacked LSTM model with the output from the tracker to identify the fall incidents. The stacked LSTM managed to effectively model the temporal information of the object’s trajectory contained in human movements. The exact instantaneous fall frame is determined using an assumption that a large movement difference with respect to the ground level along the vertical axis can be observed if a fall incident has occurred. The simulation results have proven that the proposed SmartConvFall tracker outperforms the state-of-the-art CNN-based tracker, which is also suitable to be implemented in a real-time application with less than a 1-second response time of the input video is streamed in a 30 fps format. This significant result emphasizes that our SmartConvFall has produced instantaneous response times. This could help to mitigate the slow response rate issues of the current wearable-based fall detection systems and in opposition with the current vision-based models that only focus on monitoring the presence of falls within the video sequences. Furthermore, the recent fall detection systems only manage to detect the presence of a fall after the patient has fallen and remained on the floor for a prolonged period of time. Henceforth, the short response time produced by our SmartConvFall could help to avoid any crucial fall consequences, including death and internal bleeding. The most important limitation is no existing fall detection system has been developed to specifically detect the instantaneous fall frame through the LSTM architecture. Therefore, our current work is unable to compare the performance evaluations with other related works. We have only managed to make a comparison of instantaneous fall frame detection performances between the LSTM and RNN models. The current work is also limited by the lack of publicly available fall detection datasets, particularly those that utilize the RGB camera to capture the movement of the tracked object in video sequences. Most of the fall detection datasets only provide wearable-based readings, such as accelerometer and gyroscope readings. In future works, an attention mechanism and multi-scale scheme can be integrated into the base SmartConvFall network to produce more robust exact fall frame detection.

## Figures and Tables

**Table 1:** The full configurations of the proposed tracker architecture.

Layer	Filter Size	Stride	Padding	Output	Activation Function
Conv1	7×7	2	0	96×35×35	ReLU
Pool1	3×3	2	0	96×17×17	-
Conv2	5×5	2	0	256×7×7	ReLU
Pool2	3×3	2	0	256×3×3	-
Conv3	3×3	1	0	512×1×1	ReLU
FC1	-	-	-	512	ReLU
FC2	-	-	-	512	ReLU
FC3	-	-	-	2	Softmax

**Table 2:** List of hyperparameters with their respective functions.

Hyperparameter	Type/Value	Function
Optimizer	Adam AdaGrad SGD AdaDelta RMSProp	Method to update parameters to reduce losses during training process
Learning rate	0.0001 0.001 0.01 0.1	Step size to update weights with respect to the gradient loss
Mini-batch size	64 128 256	The number of training samples for each training iteration
Number of training samples	400 600 800	Total candidate samples of $B_{train,+ve}$ and $B_{train,-ve}$

**Table 3:** List of parameters used during tracker model update.

Parameter	Value
$N_{+ve}$	50
$N_{-ve}$	200
$\sigma_{+ve}$	0.1
$\sigma_{-ve}$	2

**Table 4:** Description of different training configurations.

Training Configuration	Description
Train-1	$\left(x_{t},y_{t}\right)$
Train-2	$\left(x_{t},y_{t}\right)$ + dropout
Train-3	$\left(x_{t},|y_{t}|\right)$
Train-4	$\left(x_{t},|y_{t}|\right)$ + dropout

**Table 5:** Performance evaluations of the optimizer configurations.

Learning Rate = 0.001 Mini-Batch Size = 128 No. of Training Samples = 400										
	**Head**					**Body**				
**Type**	***EAO***	***Ac***	***Ro***	***Re***	***F_fail_***	***EAO***	***Ac***	***Ro***	***Re***	***F_fail_***
Adam	0.1668	0.4415	0.4493	0.8557	31	0.1653	0.4554	0.4493	0.8384	31
AdaGrad	0.1288	0.4377	0.9855	0.7091	68	0.1457	0.4306	0.6232	0.7799	43
SGD	0.1092	0.3964	1.0290	0.6784	71	0.1583	0.4314	0.5072	0.8132	35
AdaDelta	0.0404	0.3014	3.8382	0.2599	261	0.0637	0.2878	1.7059	0.5470	116
RMSProp	0.1543	0.4680	0.8695	0.5074	60	0.1708	0.4716	0.4928	0.8221	34

**Table 6:** Performance evaluations of the learning rate configurations.

Optimizer = Adam Mini-Batch Size = 128 No. of Training Samples = 400										
	**Head**					**Body**				
**Value**	***EAO***	***Ac***	***Ro***	***Re***	***F_fail_***	***EAO***	***Ac***	***Ro***	***Re***	***F_fail_***
0.0001	0.1669	0.4869	0.5652	0.8248	39	0.1898	0.4883	0.3623	0.8645	25
0.001	0.1651	0.4415	0.4493	0.8557	31	0.1653	0.4554	0.4492	0.8384	31
0.01	0.1693	0.4757	0.4783	0.8339	33	0.1785	0.4756	0.3913	0.8504	27
0.1	0.0788	0.4060	2.3478	0.4229	162	0.1089	0.4030	1.2319	0.6370	85

**Table 7:** Performance evaluations of the mini-batch size configurations.

Optimizer = Adam No. of Training Sample = 400 Learning Rate = 0.0001										
	**Head**					**Body**				
**Value**	***EAO***	***Ac***	***Ro***	***Re***	***F_fail_***	***EAO***	***Ac***	***Ro***	***Re***	***F_fail_***
64	0.1679	0.4719	0.5072	0.8304	35	0.1683	0.4563	0.4782	0.8268	33
128	0.1669	0.4415	0.4493	0.8557	31	0.1653	0.4554	0.4493	0.8384	31
256	0.1594	0.4633	0.4928	0.8327	34	0.1665	0.4596	0.4493	0.8349	**31**

**Table 8:** Performance evaluations of the number of training samples.

Optimizer = Adam Mini-Batch Size = 128 Learning Rate = 0.0001										
	**Head**					**Body**				
**Value**	***EAO***	***Ac***	***Ro***	***Re***	***F_fail_***	***EAO***	***Ac***	***Ro***	***Re***	***F_fail_***
400	0.1669	0.4415	0.4493	0.8557	31	0.1653	0.4554	0.4493	0.8384	31
600	0.1606	0.4462	0.5217	0.8349	36	0.1664	0.4618	0.4638	0.8277	32
800	0.1538	0.4407	0.6232	0.7930	43	0.1644	0.4581	0.4638	0.8329	32

**Table 9:** Comparison of performance evaluations for both regions of interests.

	Head	Body
*EAO*	0.1718	0.1619
*Ac*	0.4871	0.4659
*Ro*	0.4706	0.6324
*Re*	0.8274	0.7958
$F_{fail}$	32	42

**Table 10:** The average mean error of $t_{exact}$ for both stacked LSTM and RNN.

Configuration	RNN	Stacked LSTM						
		L=1	L=2	L=3	L=4	L=5	L=6	L=7
F1	36	32	29	33	32	35	30	44
F2	36	35	33	28	28	31	41	43
F3	43	36	26	29	30	32	36	37
F4	40	33	26	22	27	32	37	39

**Table 11:** Training performances using F4 configuration.

Method	Training Accuracy (%)	Training Loss	Training Duration
RNN	59.17	3.1578	1 h 4 min
Stacked LSTM:			
L=1	76.56	0.7146	2 h 44 min
L=2	91.32	0.2413	3 h 17 min
L=3	93.01	0.2112	4 h 10 min
L=4	92.05	0.2261	5 h 6 min
L=5	91.27	0.2445	5 h 36 min
L=6	90.95	0.2470	6 h 29 min
L=7	89.88	0.2666	7 h 23 min

**Table 12:** Performance comparison of each tracker for no attribute-based videos.

Tracker	*EAO*	*Ac*	*Ro*	*Re*	*F_fail_*
SmartConvFall	0.1619	0.4659	0.6323	0.7958	43
TCNN	0.1436	0.4717	0.9529	0.7062	81
MDNET-N	0.1525	0.4803	0.8941	0.7259	76

**Table 13:** Performance comparison of all tested trackers for the occlusion noise attribute.

Tracker	*EAO*	*Ac*	*Ro*	*Re*	*F_fail_*
SmartConvFall	0.1713	0.5008	0.7501	1.0833	13
TCNN	0.1249	0.4886	1.8333	0.5816	22
MDNET-N	0.0923	0.5153	2.0000	0.5118	24

**Table 14:** Performance comparison of all tested trackers for the cluttered background attribute.

Tracker	*EAO*	*Ac*	*Ro*	*Re*	*F_fail_*
SmartConvFall	0.1509	0.4813	0.5000	0.7594	2
TCNN	0.1283	0.4575	0.7500	0.6668	3
MDNET-N	0.1367	0.4341	0.7500	0.6668	3

## Data Availability

Not applicable.

## Abbreviations

The following abbreviations are used in this manuscript:

CNN	Convolutional Neural Network
RNN	Recurrent Neural Network
LSTM	Long Short-Term Memory
GPU	Graphic Processing Unit
FDD	Fall Detection Dataset
CCTV	Closed Circuit Television
IP	Internet Protocol
MEMS	Microelectrical Mechanical System
WHO	World Health Organization
MDNET	Multi-Domain Convolutional Neural Network
TCNN	Tree-structured Convolutional Neural Network
VOT	Visual Object Tracking
